# Supplementary material for: Competitiveness during Dual-Species Biofilm Formation of Fusarium oxysporum and Candida albicans and a Novel Treatment Strategy
Source: Pharmaceutics. 2022 May 30;14(6):1167. doi: 10.3390/pharmaceutics14061167 (PMC9227787; doi:10.3390/pharmaceutics14061167)
Supplement: Supplementary file 1 [file pharmaceutics-14-01167-s001.zip › pharmaceutics-1705022-supplementary.pdf]

Table S1 Name, acronym, and sequences of used primer for RT-qPCR.

**Protein names**	**Gene names**	**Primer name**	**Sequence (5′→3′)**
Agglutinin like-sequence 3	*ALS3*	*C. albicans_ALS3*_F	CTAATGCTGCTACGTATAATT
		*C. albicans_ALS3*_R	CCTGAAATTGACATGTAGCA
Ergosterol byosinthesis enzyme	*ERG11*	*C. albicans_ERG11*_F	ATTGTTGAAACTGTCATTG
		*C. albicans_ERG11*_R	CCCCTAATAATATACTGATCTG
Actin	*actin*	*C. albicans_ACT1*_F	AGCCCAATCCAAAAGAGGTATT
		*C. albicans_ACT1*_R	GCTTCGGTCAACAAAACTGG
Velvet complex subunit A	*VeA*	*F. oxysporum_VeA*_F	TTTCCCGACCTTTCTGTCCG
		*F. oxysporum_VeA*_R	CGTTTCCACCACTGTTGCTG
Velvet complex subunit B	*VelB*	*F. oxysporum_VelB*_F	CCCACCACCAGTCTCAACTC
		*F. oxysporum_VelB*_R	TTGCCCATATCCAGACTCGC
Actin-like protein arp-6	*FOYG_02810*	*F. oxysporum_FOYG_09220*_F	GGCCGACTCTACACCATCTCG
		*F. oxysporum_FOYG_09220*_R	TTTGCTTGCGACTGTCGTTG
